# Assessment of Large Language Model Performance on Medical School Essay-Style Concept Appraisal Questions: Exploratory Study

**DOI:** 10.2196/72034

**Published:** 2025-06-16

**Authors:** Seysha Mehta, Eliot N Haddad, Indira Bhavsar Burke, Alana K Majors, Rie Maeda, Sean M Burke, Abhishek Deshpande, Amy S Nowacki, Christina C Lindenmeyer, Neil Mehta

**Affiliations:** 1 Cleveland Clinic Lerner College of Medicine, School of Medicine, Case Western Reserve University, 9500 Euclid Ave, G10, Cleveland, OH, 44195, United States, 1 2164456512, 1 2164451007; 2 Department of Internal Medicine, The University of Texas Southwestern Medical Center, Dallas, TX, United States

**Keywords:** essay-type questions, large language models, generative AI, Microsoft Copilot, artificial intelligence

## Abstract

Bing Chat (subsequently renamed Microsoft Copilot)—a ChatGPT 4.0–based large language model—demonstrated comparable performance to medical students in answering essay-style concept appraisals, while assessors struggled to differentiate artificial intelligence (AI) responses from human responses. These results highlight the need to prepare students and educators for a future world of AI by fostering reflective learning practices and critical thinking.

## Introduction

Large language models (LLMs) are of growing interest in medical education. LLMs have demonstrated passing scores on the United States Medical Licensing Examination (USMLE), raising questions about their impact on assessment frameworks [1], including whether artificial intelligence (AI) can successfully answer essay-style, reasoning-based questions and whether assessors can distinguish AI-generated and student-written responses. Our medical school’s preclinical students complete application-level, essay-type questions—concept appraisals (CAPPs)—every week (Multimedia Appendix 1) [2]. We evaluated LLMs’ performance on CAPPs and examined assessors’ ability to distinguish AI-generated and human responses.

## Methods

### Study Design

Ten retired CAPP questions were selected, ensuring representation from multiple preclinical organ-system blocks, including gastroenterology, endocrinology, musculoskeletal science, cardiorespiratory medicine, hematology, renal biology, and immunology. Retired CAPPs were used, so that currently used ones were not exposed to students. Answering these required literature review and application of knowledge to clinical scenarios.

Five student responses from previous classes (before availability of LLMs) were randomly selected and deidentified. Individuals at various medical training levels generated AI responses via Bing Chat (subsequently renamed Microsoft Copilot; Multimedia Appendix 1), which used GPT-4 algorithms and had similar performance on medical tasks as ChatGPT 4.0—the most advanced LLM at the time of study [3,4]. Users first prompted Bing Chat by using the original CAPP text and then iteratively refined prompts to generate more comprehensive answers and match institutional standards without manual editing (Multimedia Appendix 1).

Ten expert assessors graded responses to 1 CAPP question each. While unaware that any responses had been AI-generated, they graded 5 deidentified student responses and 2 AI-generated responses (presented in random order) for their CAPP question, using a standard rubric (Multimedia Appendix 1). For 2 CAPPs, 4 student responses were used instead of 5 due to lack of consent for inclusion in the registry. Grading each CAPP took approximately 30 minutes; thus, a larger sample size was infeasible for this exploratory study. Afterward, assessors identified whether responses were AI- or student-generated and provided their rationales.

Scoring differences between human- and AI-generated responses and identification accuracy were evaluated, using descriptive statistics. Thematic analysis was conducted on assessors’ classification rationales; 2 team members independently analyzed reasons to identify themes, compared findings, and reconciled differences (Multimedia Appendix 1).

### Ethical Considerations

This study used deidentified data from the Cleveland Clinic Institutional Review Board–approved registry #6600. Since this was a registry for which students had already provided informed consent, separate informed consent was not required. Each CAPP reviewer was paid US $100.

## Results

**Figure 1.**
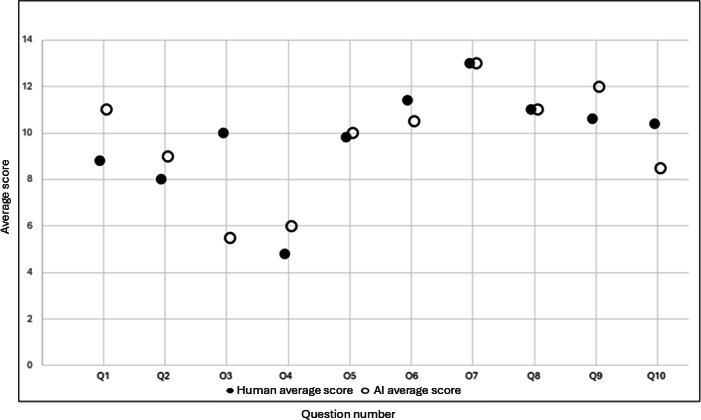
Average of human vs AI scores for each question. CAPP questions were answered either by students (human) or by prompting Microsoft Copilot (AI). Expert graders scored the CAPP questions based on a rubric. The average scores received by humans and AI are shown by question (colored vs open circles, respectively). AI responses received scores higher than or equal to those for human responses for most questions. Each question had a unique maximum score. This figure illustrates the relative scores of humans vs AI. AI: artificial intelligence; CAPP: concept appraisal.

AI responses received scores higher than or equal to those for human responses for most questions, with substantial performance variability; AI scored better than, equivalent to, or worse than humans, depending on the CAPP question (Figure 1).

Assessors correctly identified response sources 53% (36/68) of the time (student responses: 27/48, 56%; AI-generated responses: 9/20, 45%). Only 1 assessor correctly classified all responses. Consistent with other studies, 1 assessor who used AI detection tools did not have much success [5] (Table 1).

**Table 1.** Percentage of responses correctly identified as human or artificial intelligence (AI) responses for each critical appraisal (CAPP) question.^a^

Question number	Correctly identified responses, n/N (%)
Q1	3/6 (50)
Q2	3/7 (43)
Q3	3/7 (43)
Q4	6/7 (86)
Q5	3/6 (50)
Q6	2/7 (29)
Q7^b^	0/7 (0)
Q8	5/7 (71)
Q9	4/7 (58)
Q10^c^	7/7 (100)

a Responses for each question were graded by 1 expert. Expert graders were blinded and were not told which responses were generated by humans vs AI.

b Despite utilization of AI detection tools, 1 assessor did not correctly classify any of the responses (Q7).

c Only 1 assessor correctly classified all responses for their CAPP question (Q10).

Thematic analysis showed that the most cited reason for identification was the perceived “writing style,” though many assessors noted an inability to distinguish categories (Multimedia Appendix 1).

## Discussion

We demonstrate that AI can provide high-quality answers to essay-style medical education questions requiring detailed research and knowledge application. Content experts struggled to distinguish AI-generated and human-written responses, underscoring the challenges of identifying academic misuse of generative AI.

Iterative prompting of Microsoft Copilot was essential for generating acceptable responses. This process mirrors students’ typical workflow for refining drafts through edits; thus, iterative prompting does not necessarily disadvantage AI. Our findings highlight concerns about potential overreliance on AI and its implications for assessment validity, especially as recent survey data suggest that 89% of students use ChatGPT during self-study [6,7].

Given AI responses’ similarity to human responses, institutions must consider frameworks for integrating AI into assessments without compromising academic integrity [8]. Potential strategies include structured classroom use of AI during collaborative group work (eg, requiring students to assess AI responses and cite primary evidence to support or refute them) [7,9].

Study limitations include a small sample of AI-generated responses and the research’s exploratory nature. Expanding the sample size and including additional questions could provide insights on AI’s performance (relative to humans) for specific question types (Multimedia Appendix 1). Additionally, the findings prompt further discussions on ethically integrating generative AI into medical curricula while ensuring students develop critical appraisal and independent reasoning skills [7,10].

AI’s performance suggests its potential as a learning enhancement tool. However, medical educators must implement strategies for preventing overreliance on AI, fostering reflective learning practices and critical thinking, and maintaining assessment integrity.

## Supplementary material

10.2196/72034 Multimedia Appendix 1 Supplementary materials regarding concept appraisal questions and grading, Bing Chat (subsequently renamed Microsoft Copilot), the iterative prompting used in this study, and the thematic analysis.

## Abbreviations

AI: artificial intelligence
CAPP: concept appraisal
LLM: large language model
USMLE: United States Medical Licensing Examination
