# Supplementary material for: Assessment of Large Language Model Performance on Medical School Essay-Style Concept Appraisal Questions: Exploratory Study
Source: JMIR Med Educ. 2025 Jun 16;11:e72034. doi: 10.2196/72034 (PMC12208947; doi:10.2196/72034)
Supplement: Multimedia Appendix 1 [file mededu-v11-e72034-s001.docx]

# Supplement 1

## Introduction to Concept Appraisals (Essay-type) Questions

During the preclinical curriculum, students participate in problem-based learning (PBL) sessions and complete weekly concept appraisals (CAPPs). CAPPs are essay questions to confirm students thoroughly comprehend and can apply core concepts to relevant situations. Content experts provide students and their physician advisors with formative narrative feedback and complete an assessment table indicating if students fully addressed each learning objective or if they need improvement.

Some objectives are specific for a particular CAPP depending on the question topic, but 6 others are common to each assessment:

- Provided sufficient detail to reflect an appropriate depth of understanding,
- Identify and select relevant resources,
- Appropriate specific citation(s) of reference materials used,
- Logical, coherent, and well-reasoned response including grammar, spelling,
- Integrated the information into an appropriate response that covers all the concepts,
- Understood the context of the question

The use of CAPPs has proven to be an effective means to assess students’ knowledge, identify gaps, and promote self-learning. CAPP assessments combined with assessments from PBL sessions are used to document students’ performance with respect to the school’s competencies.

## Samples of CAPPs questions and grading rubrics used in the study:

### Question 1 (AI scored better than humans)

A 26-year-old woman comes to your office for an evaluation.  She was diagnosed with ulcerative colitis two years ago involving her entire colon. She asks to transfer her care over to you because of insurance issues. Her course with ulcerative colitis has been rocky over the past 8 months. She has 10 – 15 bloody bowel movements daily that are completely liquid. She has urgency to defecate and frequently is incontinent.  She reports decreased energy and weight loss of 40 lbs. Her chart shows that she has tried 5-ASAs, corticosteroids, azathioprine, and methotrexate. She says to you, “My friends who have Crohn’s disease used inflixamab and now they have their lives back! I do not want surgery. Why can’t you give me this medication?”

**1.** How does the immune response in Crohn’s disease differ from ulcerative colitis?

Please explain the cytokines and T-helper cells involved in the pathophysiology of the two diseases.

**2.** How does infliximab work?

**3.** Do you think infliximab will work for this patient?

### Grading Rubric


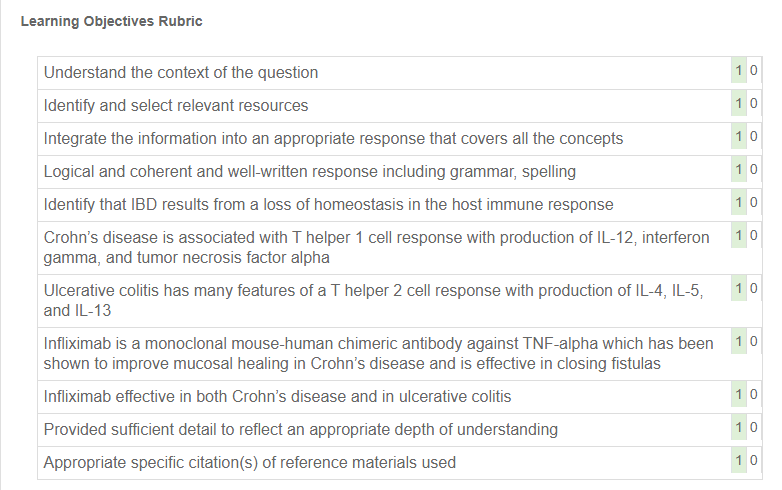


### Question 3 (Humans scored better than AI)

You are rock climbing with a friend. Your friend is 30 feet above you and falls. As she is falling past you, you reach out and are able to grab her hand. You successfully stop her fall, but her weight causes your arm to be forcefully pulled downward extending the separation/angle between your neck and your shoulder. At the same time, her arm is suddenly pulled superiorly.

Other climbers witness the event and you and your friend are rescued and taken to a hospital. However, you immediately notice that the arm you grabbed her with now hangs by your side in the adducted position, is medially rotated, and you are unable to flex your forearm at the elbow. You also have sensory deficits on the lateral aspect of your arm. Likewise, your friend also has some type of upper limb injury because she is unable to flex her fourth and fifth digits and has sensory deficits on the medial/ulnar aspect/side of her forearm and hand.

1. Explain the injury you have suffered. What is the mechanism of injury, what has been damaged, and what is the result?

2. Explain the injury that your friend has suffered. What is the mechanism of injury, what has been damaged, and what is the result?

### Grading Rubric


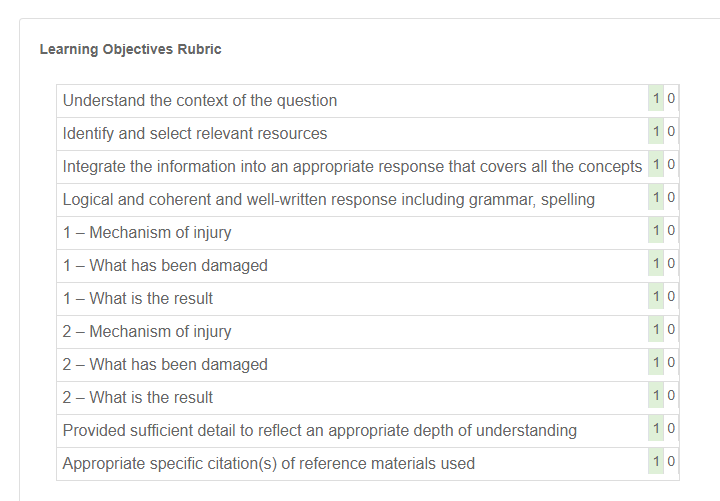


### Question 7 (Humans and AI scored comparably)

Suppose a new screening test became available for clinical use, called Fragment 1.2 (F1.2), which reflected the degree of thrombin generation, as opposed to the level of any given coagulation factor. It could therefore serve as a global coagulation test that, when elevated, would indicate hypercoagulability of the blood.

A 34-year-old male with a history of pulmonary embolism 3 years ago is referred to you for evaluation.  You find out from his medical record that he tested positive for a lupus anticoagulant at that time.  Since this is a hypercoagulable state, you now measure the amount of F1.2 generated and find that it is 1.8 times greater than expected in a normal individual.

**1.** Why would this be a better way to assess hypercoagulable potential than measuring the individual endogenous inhibitor levels (e.g. Protein C, Protein S, and Antithrombin)?

**2.** What further testing would you do to determine specifically why the F1.2 result was abnormal?

**3.** If the patient was currently receiving heparin therapy, what would the F1.2 result suggest regarding the adequacy and mechanism of heparin therapy?

**4.** You decide to recheck his lupus anticoagulant to see if it is still present. By mistake the laboratory runs the test on a sample of whole blood instead of plasma. What component of whole blood will affect the results of the lupus anticoagulant assay, how will the result be altered, and why?

**5.** If the patient had antithrombin deficiency explain the basis for an increase in thrombin generation.

### Grading Rubric


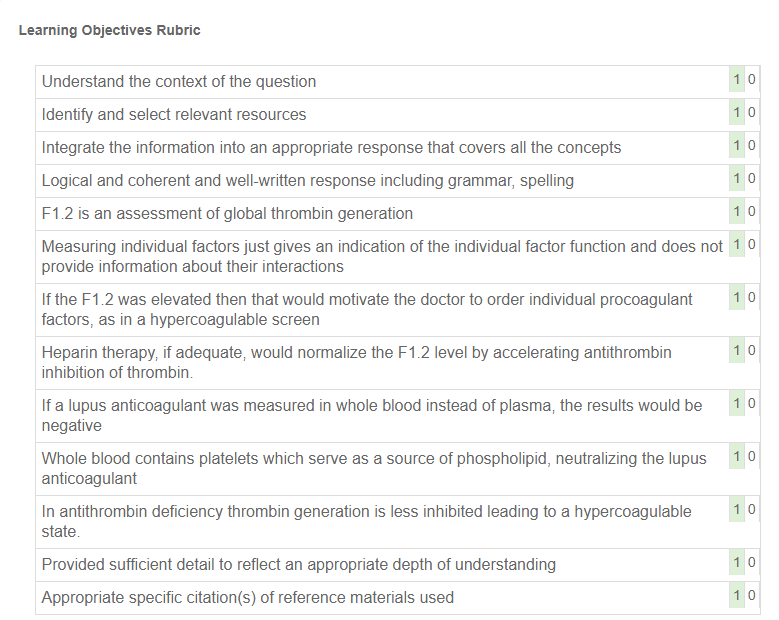


# Supplement 2

Our study was conducted in July-August 2023 and utilized Bing Chat as the Large Language Model Chatbot. At that time, Bing Chat was based on the GPT-4 algorithms utilized by ChatGPT 4.0.[1] Microsoft made Bing Chat available for free to users of the Edge Browser.[2] Unlike ChatGPT 4.0, Bing Chat also included web searching allowing users to find more current information than was available at the time when GPT-4 algorithms were developed. At the time of the study, Bing Chat was rated a top large language model based on GPT-4 algorithms.[1] Subsequently, in November of 2023, Bing Chat was rebranded as Microsoft Copilot. This rebranding was done at the same time as integration of the LLM, called Microsoft 365 Copilot, into Office 365 applications.[1]

Several studies compared the performance of ChatGPT 4.0 and Bing Chat and found minimal differences in performance on medical tasks.[3], [4]

References:

[1] “Timeline of Microsoft Copilot - Timelines.” Accessed: May 04, 2025. [Online]. Available: https://timelines.issarice.com/wiki/Timeline_of_Microsoft_Copilot

[2] “Bing Chat | Microsoft Edge.” Accessed: May 04, 2025. [Online]. Available: https://www.microsoft.com/en-us/edge/features/bing-chat

[3] K. S. Amin, M. A. Davis, R. Doshi, A. H. Haims, P. Khosla, and H. P. Forman, “Accuracy of ChatGPT, Google Bard, and Microsoft Bing for Simplifying Radiology Reports,” *Radiology*, vol. 309, no. 2, p. e232561, Nov. 2023, doi: 10.1148/radiol.232561.

[4] C. Lz *et al.*, “Performance of Generative Large Language Models on Ophthalmology Board-Style Questions,” *PubMed*, Accessed: May 04, 2025. [Online]. Available: https://pubmed.ncbi.nlm.nih.gov/37339728/

# Supplement 3

Representative example of iterative prompting used with Microsoft CoPilot.

#### Prompt 1

Entire CAPP question

#### Prompt 2

“Please expand on the above answers. Answer in a way that is in depth, accurate, and appropriate for a medical audience. Ensure that you are using sources from PubMed and that everything is referenced properly.”

#### Prompt 3

“Please make sure that all references are in order and only from PubMed.”

#### Prompt 4

“Please replace the non-PubMed sources.”

#### Prompt 5

“In text formatting is not consistent. Please ensure that it is superscript style only and that references are in order.”

# Supplement 4

Most common reasons CAPPs assessors cited when determining the author of each graded response.

# 
